# Coupling of nutrient bioavailability and nutrient ratios to microbial community structure and functional potential in lakes

**DOI:** 10.1093/ismeco/ycag150

**Published:** 2026-05-29

**Authors:** Mayra P D Rulli, Romana K Salis, Ann-Kristin Bergström, Ryan A Sponseller, Martin Berggren

**Affiliations:** Department of Physical Geography and Ecosystem Science, Lund University, Sölvegatan 12, Skåne, 223 62 Lund, Sweden; Department of Ecology and Genetics, Uppsala University, Norbyvägen 18D, Uppland, 752 36 Uppsala, Sweden; Department of Biology and Environmental Science, Linnaeus University,Universitetsplatsen 1, Småland, 392 31 Kalmar, Sweden; Department of Ecology, Environment and Geoscience, Umeå University, Linnaeus väg 6, Västerbotten, 901 87 Umeå, Sweden; Department of Ecology, Environment and Geoscience, Umeå University, Linnaeus väg 6, Västerbotten, 901 87 Umeå, Sweden; Department of Physical Geography and Ecosystem Science, Lund University, Sölvegatan 12, Skåne, 223 62 Lund, Sweden

**Keywords:** relative nutrient availability, DOM bioavailability, nutrient ratios, metabolic functional diversity, aquatic biogeochemistry, DOC, carbon, nitrogen, phosphorus, nutrient bioavailability

## Abstract

Microbial communities play a fundamental role in lake nutrient cycling, yet their composition and functional diversity in response to environmental gradients remain poorly understood. Specifically, little is known about how the supply of dissolved macronutrients, including inorganic and bioavailable organic fractions, shape microbial community structure, and functional diversity in lakes that are strongly subsidized by terrestrial inputs. Boreal lakes, with varying concentrations of total and bioavailable dissolved organic carbon (DOC), nitrogen (N) and phosphorus (P), provide an ideal setting to investigate these dynamics. Here, we hypothesize that microbial pathways related to N and P acquisition, as inferred from marker-gene data, are more represented under relative deficiency of available N and P resources, respectively. To test this, we analysed the rRNA-inferred microbial community composition and metabolic functional diversity across 34 south-Swedish lake outlets in relation to bioavailable nutrient supply. Results show that DOC and P were key drivers of microbial community structure, with bulk DOC concentrations being most relevant for bacteria (16S rRNA), while bioavailable fractions of DOC and P were relatively more influential for eukaryotic communities (18S rRNA). Predicted N- and P-related metabolic pathways correlated with nutrient ratio imbalances, supporting our hypothesis that microbial communities adjust their metabolic strategies in response to relative nutrient demand. These findings demonstrate that accounting for nutrient ratios and bioavailability, in addition to bulk concentrations, helps provide an improved mechanistical understanding of microbial functional potentials in lakes.

## Introduction

Microbial communities play a central role in the biogeochemical cycles of carbon (C), nitrogen (N), and phosphorus (P) in freshwater ecosystems [1, 2]. Through processes such as assimilation, transformation, and excretion, microbes interact with and reshape the nutrient pools, thereby influencing ecosystem productivity and stability [3]. These microbial processes are dependent upon the supply and quality of macronutrient resources, including organic substrates [4, 5]. Understanding how microbial communities respond to shifts in resources is therefore crucial for predicting ecosystem functioning, especially in regions undergoing rapid environmental change [5].

One of the greatest challenges in studies of microbial nutrient responses is that commonly measured bulk nutrient fractions poorly represent what is biologically accessible to microorganisms [6–8]. Dissolved inorganic N (DIN) and soluble reactive P (SRP; mainly phosphate) are often used as proxies for bioavailable N and P, respectively, overlooking the key role of additional organic nutrient compounds in supporting microbial metabolism [9, 10]. Bulk dissolved organic carbon (DOC) is widely measured as a proxy for organic C resources, but the amount of directly bioavailable C is a small and highly variable part of DOC [11, 12]. Yet bioavailable macronutrient fractions are critical for regulating the growth and composition of microbial communities [2, 13]. Despite their ecological importance, we still lack a clear understanding of how bioavailable pools of C, N, and P jointly shape microbial nutrient acquisition and recycling strategies, particularly for N and P, where microbes access nutrients via uptake of inorganic forms or enzymatic breakdown of organic forms and subsequent uptake, while also conserving nutrients intracellularly under limitation or releasing excess through excretion [14, 15].

For nearly a century, nutrient availability has been central to explaining ecosystem processes in aquatic research (e.g. [16]), leading to broadly applicable frameworks such as the “Redfield ratio” approach to assess nutrient limitation of primary production from concentrations of inorganic N and P [17]. However, no corresponding framework exists to explain microbial metabolism, which also relies on dissolved organic matter (DOM) as a source of bioavailable N and P, and as a source of energy and C. The microbial use of DOM depends strongly on its quality, governing the bioavailability of associated C, N, and P pools [18, 19]. Little is known about how imbalances among total bioavailable macronutrient resources, including bioavailable DOM, influence microbial community composition, and nutrient acquisition pathways (e.g. [20, 21]). It can, however, be expected that nutrient resource responses differ across main functional taxonomic groups of bacteria and microbial eukaryotes, including osmotrophs, phototrophs, and mixotrophs, which differ in their modes of metabolism.

In this study, we investigated how C, N, and P ratios as well as bioavailable and commonly measured fractions of C, N, and P relate to microbial community composition and predicted functional potential in boreal lakes. By integrating direct measurements of nutrient bioavailability with gene marker data across 34 south-Swedish lakes, we aimed to improve understanding of microbial nutrient utilization strategies in natural systems. Specifically, we hypothesized that: (i) microbial functional pathways related to C, N, and P metabolism are increasingly represented in response to relative deficiencies of bioavailable macronutrients, reflecting metabolic adjustments to relative nutrient availability; and (ii) bacterial and eukaryotic communities exhibit distinct responses to bioavailable nutrients, reflecting differences in metabolic strategies, such as osmotrophy, photoautotrophy, and mixotrophy.

## Materials and methods

### Sites and sampling

**Figure 1:**
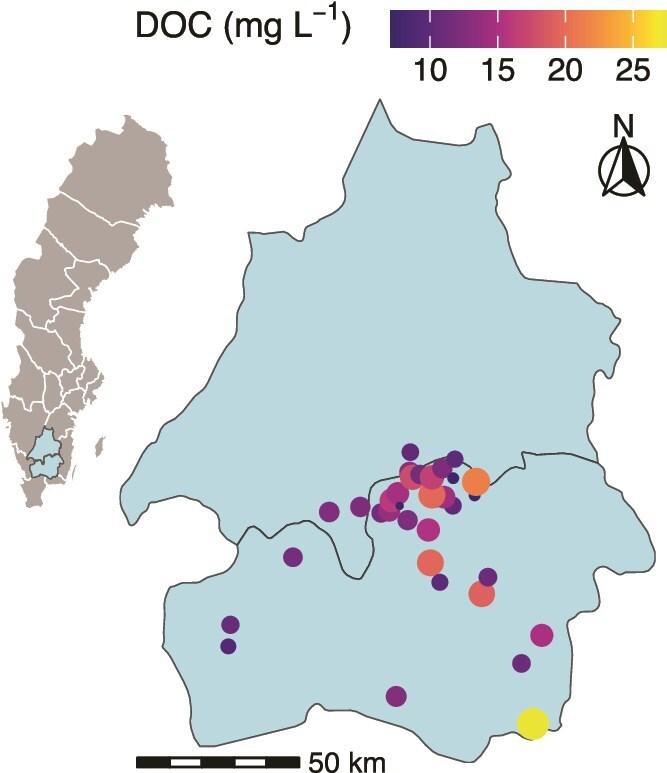
The locations of the 34 sampled lakes in the county of Småland, Sweden. Lakes are represented by circles, with sizes and colours scaled by DOC concentration among the study sites. Locations refer to the outlet streams sampled, representing each lake’s position.

We collected water samples representing 34 lakes in the Kronoberg and Jönköping counties in the Småland region of Southern Sweden on 26–27 July 2021 (Fig. 1). Samples were collected from the outlet stream of each selected lake (Table S1), which area ranged from 20 to 17 300 ha (Table 1). During summer stratification, lake outlets typically draw water from the epilimnion, the warm and well-mixed surface layer where most biological activity occurs [22]. As such, outlet sampling provides an integrated signal of epilimnetic water conditions. We note that this approach assumes that outlets primarily capture surface waters rather than deeper layers, which may vary depending on lake morphometry and outlet structure.

**Table 1:** Summary [mean ± standard deviation (SD) and median with interquartile range (IQR)] of lake characteristics, bioavailable and total or inorganic nutrient concentrations, and nutrient ratios for the 34 study lakes.

	Mean (± SD)	Median (IQR)
*Lake characteristics*		
Lake size (ha)	1073 (± 3104)	100 (40–312)
*Concentrations*		
Water colour (mg Pt L^−1^)	83.62 (± 49.09)	65.5 (55–106)
SUVA (L mg^−1^ m^−1^)	2.44 (± 0.51)	2.31 (2.12–2.78)
DOC (mg L^−1^)	13.38 (± 4.38)	12.38 (10.45–15.21)
NO_3_ + NO_2_ (μg L^−1^)	105 (± 251)	26 (9.85–66.25)
NH_4_ (μg L^−1^)	114 (± 363)	32.9 (25.75–60.2)
DIN (μg L^−1^)	219 (± 442)	61.8 (46.18–150)
SRP (μg L^−1^)	5.68 (± 7.20)	4 (2.95–4.8)
BDOC (μg L^−1^)	4773 (± 4433)	3723 (2226–5185)
BTDN (μg L^−1^)	321 (± 402)	164 (103–273)
BTDP (μg L^−1^)	4.54 (± 7.18)	2.15 (1.51–5.47)
Chl-*a* (μg L^−1^)	3.05 (± 3.19)	1.84 (1.14–3.78)
*Ratios*		
DIN:SRP	76.94 (± 104)	45.97 (24.4–74.7)
DOC:SRP	8988 (± 4470)	8691 (5574–11 453)
DOC:DIN	228 (± 158)	188 (119–333)
BTDN:BTDP	321 (± 415)	175 (90.95–287)
BDOC:BTDP	9099 (± 10 823)	4606 (3512–9282)
BDOC:BTDN	40.7 (± 48.25)	24.01 (11.03–54.34)

SD = standard deviation; IQR = interquartile range (25th–75th percentile)

Water was collected from each site in 1 L polypropylene bottles (a bottle per site) by gently submerging bottles just below the surface, typically near the centre of the outlet stream where flow is greatest and rinsing them with site water prior to collection. Outlet streams are generally well mixed due to flow and turbulence [23], and thus provide a representative sample of surface waters, although mixing conditions may vary among sites. Samples were then stored in the dark and kept cool (~4°C) until further processing in the laboratory (within 24–48 h). We also filtered between 120 and 420 ml (depending on particulate content and filter clogging) of water from each stream through 0.22 μm Millipore® Sterivex™ pressure filters using sterile 60 ml syringes. After filtration, the water was discarded, and the filters were stored cold and dark in sterile 50 ml Falcon® tubes until arriving in the laboratory, where they were stored in the freezer at ~−80°C for subsequent DNA extraction and sequencing.

### Sample processing, nutrient, and dissolved organic matter analysis

Within 48 h of sampling, water was filtered through ~0.7 μm GF/F microfiber filters (Whatman) followed by 0.2 μm nylon membrane filters (Whatman) to remove most bacterial cells. The GF/F filters were used for chlorophyll-*a* (chl-*a*) analysis, and 0.2 μm filtered water was either stored cold (~4°C) for DOM analysis (10 ml Aqualog vials) or frozen (−18°C) for nutrient analysis (50 ml Falcon® tubes). Freshly filtered water was also used for bioassay preparation.

Chl-*a* was extracted in 95% ethanol for 24 h in the dark with intermittent shaking, then measured on a fluorometer (PerkinElmer LS-55; excitation: 433 nm, emission: 672 nm) calibrated with *Anacystis nidulans* reference material (Sigma Aldrich) and with a quantification limit lower than 0.2 μg L^−1^. Subsamples for DOC were stored cold (~4°C) in acid-washed 40 ml amber vials, acidified to pH 2 with 10% HCl, and analysed at the Jan Veizer Stable Isotope Laboratory (University of Ottawa).

Dissolved NO_2_ + NO_3_, NH_4_, and PO_4_ were analysed using a QuAAtro 39 continuous flow analyser (Seal Analytical) with colourimetric detection: NO_2_ + NO_3_ by cadmium reduction coil (MT3B Q-126-12 Rev 1), NH4 by the salicylate method (Q-033-04 Rev. 8), and PO_4_ by the molybdenum blue method (MT3A Q-125-12 Rev 1). DIN was calculated as the sum of NO_2_-N, NO_3_-N, and NH_4_-N. Limits of quantification were ~0.07, ~0.4, and ~ 0.3 μg L^−1^ for NO₃-N, NH₄-N, and PO₄-P, respectively.

DOM characteristics were assessed using Aqualog absorbance spectra (HORIBA; 230–800 nm, 1 cm quartz cuvette). Absorbance was corrected using Milli-Q blanks and converted to Napierian absorption coefficients (m^−1^). Water colour was estimated from absorbance at 440 nm following [24], and specific ultraviolet absorbance (SUVA) was calculated as absorbance at 254 nm (m^−1^) divided by DOC (mg L^−1^), yielding values in L mg^−1^ m^−1^ as a proxy for DOM aromaticity.

### Bioavailability bioassays and bioavailable resource and ratios estimation

We conducted standardized microbial bioassays to estimate the bioavailable fractions of DOC (BDOC), total dissolved N (BTDN), and total dissolved P (BTDP) across lakes. A pooled microbial inoculum (2% v/v) was prepared by combining water from all lakes, filtered through ~1.2 μm GF/C microfiber filters (Whatman) to remove large particles and large bacterivores. This ensured consistent microbial composition across assays, isolating the effects of macronutrient chemistry on bioavailability.

Assays followed a modified bacterial regrowth protocol based on [7], adapted to a 96-well plate format for high-throughput analysis [25]. Filtered lake water was supplemented with a micronutrient growth medium and macronutrients (combinations of 20 mg C L^−1^ as C_6_H_12_O•H_2_O, 2000 μg N L^−1^ as NH_4_NO_3_, and 200 μg P L^−1^ as Na_2_HPO_4_•2H_2_O depending on the targeted macronutrient) to induce strong C, N, or P limitation and, in parallel, we had wells spiked with the limiting nutrient (1000 μg C L^−1^; 100 μg N L^−1^; 10 μg P L^−1^ for C-, N- or P-limited incubations, respectively). Here, we assume that limitation is achieved and all the added spike is consumed, thus resulting in successful bioavailability assay. Plates were incubated for 72 h in the dark at 20°C. Bacterial cell counts were measured by flow cytometry at 24 h intervals, with estimates of BDOC, BTDN, and BTDP based on net bacterial cell yields and nutrient assimilation per cell. The spike concentration of each incubation group (C, N, or P) divided by the difference between the cell abundance from spiked and non-spiked incubations were used to calculate nutrient assimilation per bacterial cell. If the difference between the cell abundance from spiked and non-spiked incubations result in a negative value, the bioassay is considered unsuccessful as the limitation assumption was not fulfilled, and thus the respective sample is removed from all further analysis and considered as a missing value (Table S2). The nutrient assimilation per bacterial cell was then multiplied by the cell abundance of non-spiked incubations to get an estimate of the bioavailable DOC, TDN, and TDP concentrations from their respective incubations. Full methodological details are provided in the Supplementary Information.

To assess relative nutrient availability and potential elemental imbalances, C:N:P ratios were calculated using molar concentrations of DOC, DIN, and SRP, alongside their bioavailable fractions. These ratios were compared to Redfield benchmarks (106:16:1), originally derived from marine phytoplankton [17], and the reported stoichiometric ranges for heterotrophic freshwater bacteria [26], which are typically more variable than Redfield values. Log-transformation was applied to all ratios prior to statistical analysis, following best practices in ecological stoichiometry [27].

### Microbial community analysis

To characterize microbial community composition and enable downstream inference of functional potential, we performed 16S and 18S rRNA gene amplicon sequencing. We extracted total DNA from water samples using the MO BIO PowerSoil DNA isolation kit (MO BIO Laboratories), following the manufacturer’s protocol with modifications for Sterivex™ filters to optimize cell lysis and DNA recovery from low-biomass aquatic samples. To ensure accurate measurement of double-stranded DNA prior to library preparation, we performed DNA quantification following the protocol by [28] using a Qubit fluorometer. Amplicon sequencing of the 16S (V6–V8) and 18S (V4) rRNA gene regions was conducted at the Integrated Microbiome Resource in Halifax, Canada, using an Illumina MiSeq platform with paired-end 2 × 300 bp reads. These marker-gene regions were selected to characterize bacterial and microbial eukaryotic community composition, respectively, using primers B969F/BA1406R (16S) and E572F/E1009R (18S) [29].

To generate high-resolution microbial community composition data for downstream ecological and statistical analyses, raw demultiplexed sequences were processed in R using the DADA2 pipeline [30], which infers exact Amplicon Sequence Variants (ASVs) and reduces sequencing error. Taxonomic classification was assigned using the SILVA (v138.1) and PR2 (v5.0.0) database for 16S and 18S rRNA data, respectively, to enable classification of bacterial and microbial eukaryotic taxa. We used the “phyloseq” package [31] to filter sequences and retain only bacterial and microbial eukaryotic taxa. ASVs annotated as chloroplasts (order level) or mitochondria (family level) were excluded from the 16S dataset to remove non-target and host-derived sequences. Final ASV tables contained 1 286 321 16S sequences (13 102 ASVs) and 1 779 140 18S sequences (11 585 ASVs). Full sequencing statistics are described in the Supplementary Information and Table S3, and rarefaction curves are provided in Fig. S1.

To assess the potential metabolic capabilities of bacterial communities in relation to nutrient availability, bacterial functional potential was predicted using PICRUSt2 (v2.5.2) [32], which infers gene and pathway abundances from 16S rRNA gene ASVs based on phylogenetic placement. We then used KEGG Orthology identifiers to annotate metabolic pathways. As these predictions represent putative functional potential rather than direct measurements of gene expression or activity, results were interpreted with this limitation in mind. Pathways were analysed at two levels: (i) specific pathways, representing individual KEGG pathways related to C, N, P metabolism or iron (Fe)/metal acquisition; and (ii) general pathways, calculated as the summed relative abundance of all specific pathways within each of these four categories to enable comparison of broader functional responses.

To complement functional predictions and provide a trait-based perspective on microbial community responses, we attributed trophic groups (phototrophs, heterotrophs, mixotrophs) to ASVs based on their assigned taxonomy using the Mixoplankton Database [33] and higher-level taxonomic classification (Table S4). This classification allowed us to examine whether shifts in community composition correspond to differences in dominant nutritional strategies across environmental gradients. However, these classifications are coarse and may not capture intragroup variability, as not all taxa within a given group (e.g. mixotrophs) necessarily express the assigned trophic strategy [33], underscoring the need for finer taxonomic resolution in trait databases.

Detailed protocols for microbial community analysis and bioinformatics processing are provided in the Supplementary Information.

### Statistical analysis

All statistical analyses were conducted in R. To evaluate relationships between nutrient availability and nutrient ratios imbalances, we applied a combination of regression-based and non-parametric approaches. Relationships between nutrient concentrations (e.g. BDOC vs. DOC) and their ratios (e.g. BDOC:BTDN vs. DOC:DIN) were assessed using linear regression where assumptions of normality and homoscedasticity were met, and Spearman rank correlations otherwise. For pairwise comparisons among groups, Dunn’s tests were applied with false discovery rate (FDR) correction. Significance was set at α = 0.05.

To assess how nutrient availability and nutrient ratios imbalance structure microbial community composition, multivariate analyses were conducted using distance-based approaches. We calculated community dissimilarities using Aitchison distance following centred log-ratio (CLR) transformation to account for the compositional nature of amplicon data. To evaluate relationships between community composition and nutrient variables, we used distance-based redundancy analysis as the primary constrained ordination method. Separate models were constructed for C- (total DOC and bioavailable DOC), N- (inorganic N and bioavailable TDN), and P-related (inorganic P and bioavailable TDP) variables, with environmental variables centred (CLR) and scaled prior to analysis.

Finally, to assess potential functional and trophic responses to relative nutrient availability (hypothesis 1), Spearman rank correlations were calculated between the relative abundance of predicted pathways or trophic groups and environmental variables, including log-transformed nutrient ratios. We conducted analyses for bacterial and eukaryotic communities separately to evaluate differences in responses between microbial groups (hypothesis 2). FDR correction was applied to account for multiple testing. Full model specifications, R packages, and visualization details are provided in the Supplementary Information.

## Results

### Biogeochemical conditions and lake characteristics

Nutrient concentrations and lake characteristics spanned broad gradients (Table 1). DOC concentrations were generally high (mean ± SD: 13.38 ± 4.38 mg L^−1^), while DIN and SRP concentrations showed substantial variability across lakes (range: 24–2209 and 1.8–41 μg L^−1^, respectively; Table S2). Similarly, the concentrations of bioavailable nutrient fractions varied widely among sites, with BDOC, BTDN, and BTDP ranges of 1.3–22 mg L^−1^, 36–1482 μg L^−1^, and 0.26–41 μg L^−1^, respectively (Table S2). SUVA ranged from 1.5 to 3.7 L mg^−1^ m^−1^, with 5 out of the 34 sampled lakes with values below 2 L mg^−1^ m^−1^ (Table S1). The remaining lakes had SUVA values ranging from 2 to 4 L mg^−1^ m^−1^.

**Figure 2:**
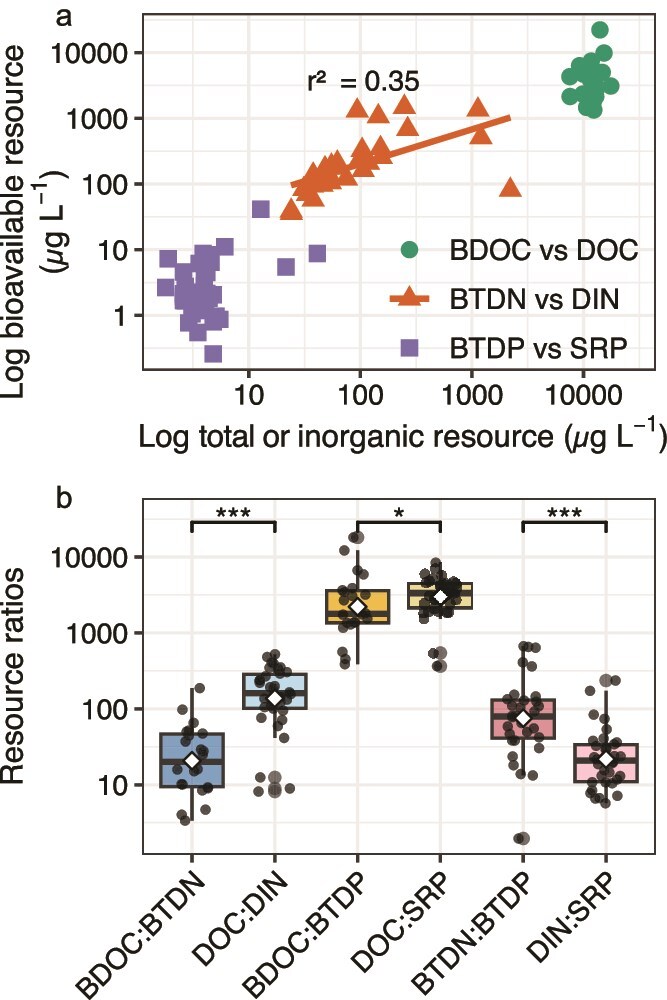
Relationships between total or inorganic and bioavailable concentrations of C, N, and P. (a) Scatter plot showing the relationship between log-transformed bioavailable and total concentrations of C (circles), inorganic N (triangles) and P (squares) across the 34 study lakes. A linear regression line with the corresponding r^2^ value was added when the relationship between bioavailable and total or inorganic concentrations was significant (*P* < .05). (b) Boxplots displaying bioavailable and total or inorganic C:N, C:P, and N:P ratios (y-axis is presented in log10-scale). The median and individual data points are shown, with mean points overlaid as white diamonds. Asterisks above the boxplots indicate the following significance levels: ^***^ for *P* < .001, ^*^ for *P* = .05.

The relationship between bioavailable and total or inorganic fractions varied among C, N, and P (Fig. 2A). A weak yet significant positive correlation was observed between BTDN and DIN (*P* < .001, r^2^ = 0.35). In contrast, relationships between bioavailable and total DOC, as well as BTDP and SRP, were not significant (*P* > .05, r^2^ = 0.04 and 0.12, respectively).

Nutrient availability ratios were highly variable, with BTDN:BTDP ratios higher than DIN:SRP, while BDOC:BTDN and BDOC:BTDP ratios were comparatively lower than DOC:DIN and DOC:SRP, respectively (Fig. 2B, Table 1). Pairwise comparisons revealed that the BDOC:BTDN and BTDN:BTDP ratios were significantly different from their total or inorganic counterparts (*P* < .001), while the difference in C:P ratios was only marginally significant (*P* = .05; Fig. 2B).

Across lakes, DOC:DIN ratios were well above both the Redfield threshold of 6.6 and the Godwin–Cotner range of ~4–7 [26] (range: 9.5–610; Table S5), and BDOC:BTDN ratios in 86% of the studied lakes were also above these threshold (mean ± SD: 40.7 ± 48.3, *n* = 22). Conversely, 91% and 97% of the sites had DIN:SRP and BTDN:BTDP ratios, respectively, higher than the Redfield ratio of 16 (Table S5). Similarly, DOC:SRP and BDOC:BTDP ratios greatly exceeded the Redfield C:P threshold of 106 (ranges: 941–21 634 and 998–46 684, respectively). Interestingly, 3 out of the 32 lakes (9%) had BTDN:BTDP higher than the upper range (i.e. >1200) reported by [26] for heterotrophic bacteria in lakes.

### Microbial predicted functional pathways and stoichiometric constraints

**Figure 3:**
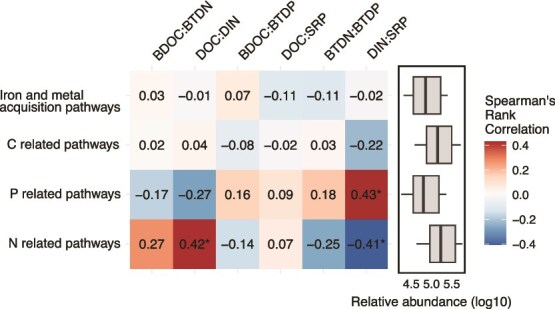
Heatmap of the Spearman’s rank correlations displaying the correlation coefficient values (*rho*) between nutrient ratios (BDOC:BTDN, DOC:DIN, BDOC:BTDP, DOC:SRP, BTDN:BTDP, and DIN:SRP) and general predicted functional pathways. Cell shading indicates the strength and direction of the correlation (red = positive, blue = negative), with asterisks indicating statistical significance (^*^P < .05, not FDR-adjusted). The boxplot shows the log10-transformed abundance for each functional pathway.

Based on PICRUSt2 predictions of functional potential derived from 16S rRNA gene data, we assessed the predicted relative abundance of metabolic pathways involved in nutrient cycling across lake samples. General P-related pathways (Fig. 3) were positively correlated with DIN:SRP ratios (*P* = .01), suggesting that P metabolism intensifies with higher DIN:SRP ratios but is reduced when P is in excess relative to N. These pathways accounted for 0.3% of the total metabolic pathways and 15.5% of the studied pathways (Fig. S2). Nitrogen-related pathways were more prevalent, comprising 0.6% of the total and 37% of the studied pathways, and showed positive correlations with DOC:DIN (*P* = .01, *rho* = 0.42) and negative correlations with DIN:SRP (*P* = .02, *rho* = −0.41), consistent with a greater functional emphasis on N metabolism under C-rich and/or N-poor conditions (Fig. 3). Additionally, general N-related pathways were negatively correlated with both BTDN and DIN (*P* = .03, *rho* = −0.39 and −0.37, respectively; Fig. S3). Carbon-related and iron/metal acquisition pathways did not show significant correlations with the stoichiometric ratios tested but are further explored below.

**Figure 4:**
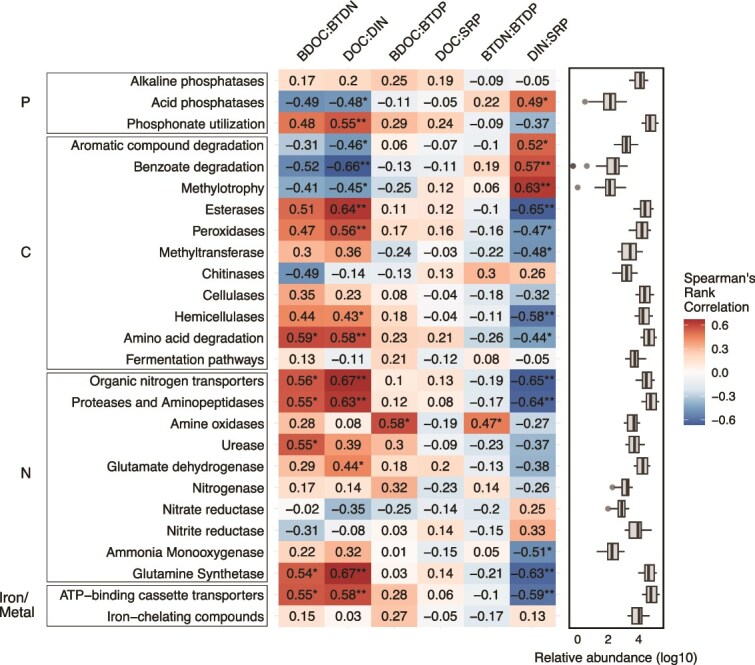
Heatmap of the Spearman’s rank correlations displaying the correlation coefficient values (*rho*) between the top specific predicted functional pathways and nutrient ratios (BDOC:BTDN, DOC:DIN, BDOC:BTDP, DOC:SRP, BTDN:BTDP, and DIN:SRP). Cell shading indicates the strength and direction of the correlation (red = positive, blue = negative), with asterisks indicating statistical significance (^*^P < .05, ^**^P < .01, FDR-adjusted). The boxplot shows the log10-transformed abundance for each specific functional pathway.

Building on the patterns observed for general pathways (see Figs 3, S2–S3), specific predicted functional pathways exhibited distinct correlations with nutrient ratios (Fig. 4). Notably, while C-related pathways, which accounted for 0.5% of total metabolic pathways and 32% of the studied ones, showed no significant relationships at the general level, several specific C-degradation pathways were significantly correlated with DOC:DIN and DIN:SRP. Pathways positively correlated with DOC:DIN were negatively correlated with DIN:SRP (e.g. esterases pathway; *P* = .001), while those negatively associated with DOC:DIN showed positive correlations with DIN:SRP (e.g. benzoate degradation pathway, *P* = .001 and .006, respectively; Fig. 4). Similarly, key N-related pathways, including organic N acquisition and processing, were positively correlated with BDOC:BTDN and DOC:DIN, and negatively with DIN:SRP (e.g. organic N transporters pathway; *P* = .043, .001, and .001, respectively; Fig. 4). Moreover, P-related pathways were linked to DOC:DIN as well as DIN:SRP, with acid phosphatases increasing at high N:P (*P* = .03; Fig. 4) and low C:N ratios (*P* = .03; Fig. 4), while phosphonate utilization pathway increased with C:N ratios only (*P* = .008; Fig. 4).

Additional correlations with inorganic and bioavailable nutrients (Fig. S4) revealed that increased N availability (BTDN, DIN) was negatively correlated with organic N processing pathways (*P* = .01 and .004, and *rho* = −0.57 and −0.63, respectively), while BTDP was positively correlated with C-degradation pathways such as benzoate degradation (*P* = .03, *rho* = 0.5) and methylotrophy (*P* = .01, *rho* = 0.57).

### Microbial community responses to nutrient imbalances

**Figure 5:**
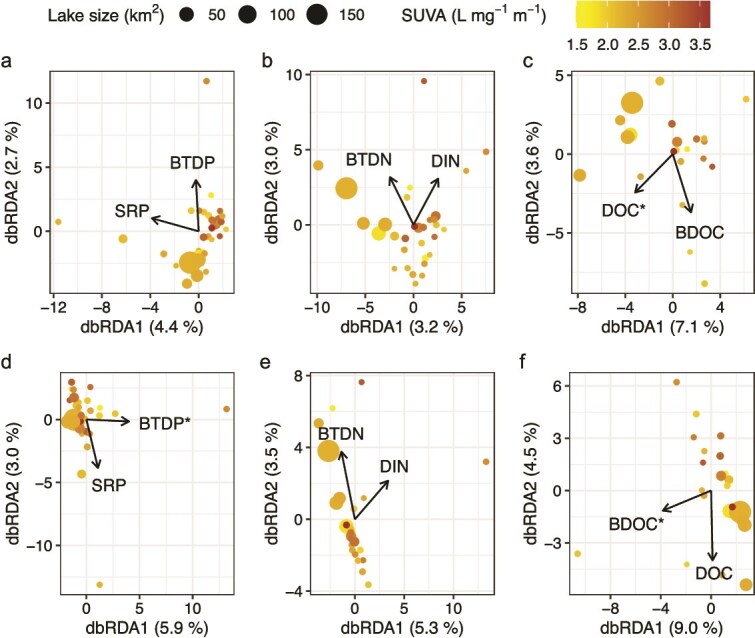
Redundancy analysis examining the relationship between microbial community composition based on 16S rRNA (A–C; bacterial communities) and 18S rRNA (D–F; eukaryotic communities) amplicon sequencing and key total or inorganic and bioavailable nutrients in lake water samples. Lake size is represented by point sizes, and SUVA (L mg^−1^ m^−1^) is depicted with a colour gradient. The panels focus on total or inorganic and bioavailable fractions of (A) and (D) phosphorus (SRP and BTDP), (B) and (E) nitrogen (DIN and BTDN), and (C) and (F) carbon (DOC and BDOC).

Redundancy analysis revealed distinct microbial community responses to nutrient variables, although the total variation explained by these models was modest. For bacterial communities, total DOC significantly structured composition, explaining 57% of the constrained variance (*P* = .049, F = 1.369; Table S6, Fig. 5C). In contrast, eukaryotic communities showed stronger associations with bioavailable nutrient fractions, with BTDP (*P* = .036, F = 1.866) and BDOC (*P* = .001, F = 1.967) significantly contributing to community shifts and explaining 66% and 65% of the constrained variance, respectively (Table S6).

### Eukaryotic trophic groups and nutrient dynamics

**Figure 6:**
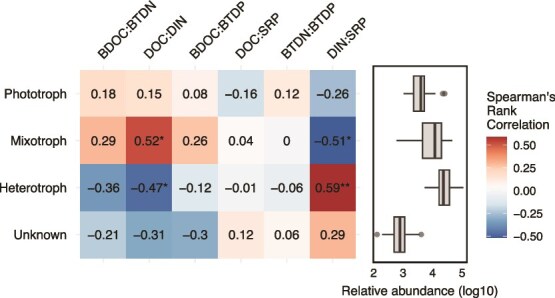
Heatmap of the Spearman’s rank correlations displaying the correlation coefficient values (*rho*) between trophic group (phototroph, mixotroph, heterotroph, unknown) and nutrient ratios (BDOC:BTDN, DOC:DIN, BDOC:BTDP, DOC:SRP, BTDN:BTDP, and DIN:SRP). Cell shading indicates the strength and direction of the correlation (red = positive, blue = negative), with asterisks indicating statistical significance (^*^P < .05, ^**^P < .01, FDR-adjusted). The boxplot shows the transformed abundance for each trophic group.

Relative abundance data showed that heterotrophic and mixotrophic taxa dominated eukaryotic microbial communities across lakes (mean ± SD: 58 ± 23% and 29 ± 21%, respectively; Fig. S5). The Spearman correlation revealed distinct trophic group responses to nutrient ratios (Fig. 6). The abundance of mixotroph marker-genes increased during N limitation, as shown by its positive correlation with DOC:DIN (*P* = .02) but negative correlation with DIN:SRP (*P* = .02). Heterotrophs exhibited the opposite pattern, showing a negative correlation with DOC:DIN (*P* = .03) and a positive one with DIN:SRP (*P* = .008). Trends were also observed in corresponding correlations with bioavailable and inorganic nutrient variables (Fig. S6), where mixotrophs were negatively correlated with BTDP (*P* = .02, *rho* = −0.53) and DIN (*P* = .005, *rho* = −0.61), while heterotrophs showed positive correlations with these same resources (*P* = .03 and .02, and *rho* = 0.5 and 0.54, respectively). No significant correlations were observed for phototrophs.

## Discussion

This study provides new insight into how nutrient gradients shape distinct microbial responses in boreal freshwater ecosystems. Our findings build on previous work linking relative nutrient availability to microbial processes [5, 16, 34], while expanding this knowledge by taking inferred microbial functional pathways into account, and by considering both bioavailable and commonly measured nutrient fractions. As land use and climate continue to alter nutrient inputs and organic matter quantity and quality, understanding these dynamics is essential for predicting the role of microbes in freshwater biogeochemical cycles [35–37]. While PICRUSt2-based functional inference provides valuable insights into potential metabolic patterns, these predictions remain constrained by the availability and quality of reference genomes and annotations, potential biases related to 16S rRNA gene copy number variation, and the fact that they reflect genetic potential rather than active metabolic processes [32, 38]. Future studies integrating metagenomic and metatranscriptomic approaches would provide more direct insights into microbial activity and help validate the functional patterns observed.

### Nutrient ratios imbalances shape nitrogen and carbon metabolism

Nitrogen is a key limiting nutrient in many northern freshwater ecosystems [39, 40], and microbial communities employ diverse metabolic strategies to optimize its acquisition under variable nutrient ratios conditions [16]. In our study, both C- and N-related predicted pathways were more represented under high DOC:DIN and low DIN:SRP ratios (Fig. 4), indicating response to N scarcity. While one might expect increased representation of C-related pathways under C limitation [41, 42], the high observed DOC concentrations (Table 1) and weak association between BDOC and bacterial communities (Fig. 5C) appear to weaken such connections. Instead, microbes appeared to invest in C metabolism, particularly degradation of hemicellulose, lignin, and aromatic compounds, to access N embedded in complex organic matter [41, 42]. These shifts align with microbial responses to C-rich, N-limited environments, including greater use of proteolytic enzymes, amino acid transport [43], and ammonium assimilation or N fixation [44–46]. As N availability increases, microbial communities may reduce investment in organic N acquisition and shift toward other nutrient strategies, such as P uptake [44, 47].

The type of C substrates targeted by microbial communities also shifted with nutrient availability. Predicted pathways involved in degrading simple compounds (e.g. benzoate, methylated compounds) were associated with low DOC:DIN and high DIN:SRP conditions, suggesting that labile C degradation is favoured when N is more available, while complex organic matter degradation is prioritized under N-limited conditions. These findings highlight how microbial C and N metabolism are tightly coupled and modulated by nutrient ratios constraints [41, 47, 48].

The stronger association between N-related predicted pathways and inorganic nutrients rather than bioavailable pools suggests that long-term nutrient ratios imbalances may influence microbial N metabolism more strongly than short-term fluctuations in labile organic N supply [34, 49, 50]. Our bioassays estimated resource bioavailability over 48 h, representing a short-term pulse of labile nutrients. While inorganic nutrients can also fluctuate on short timescales [51, 52], their stronger correlations imply they may provide more reliable signal of N availability. Nevertheless, specific N-related pathways, such as urease and organic N transporters, increased with BDOC:BTDN ratios (Fig. 4), indicating microbial sensitivity to DOM composition. This is consistent with observations that microbes prioritize labile organic N when inorganic N is scarce [34, 49] and rapidly process amino acids when DOM provides accessible N and C [49, 50]. As proposed by Lutz *et al.* [53], amino acids may serve dual roles depending on context, functioning as an energy source under C limitation or a nitrogen source when N is scarce. Our findings support the latter, where amino acid processing reflects microbial adaptation to N limitation under C-rich conditions.

Predicted pathways involved in recalcitrant organic matter breakdown, such as peroxidases and esterases, were negatively correlated with DIN and BTDP (Fig. S4), suggesting that higher inorganic nutrient availability may reduce microbial investment in complex organic matter degradation [54, 55]. Although we did not measure gene expression directly, this finding agrees with previous observations that microbes downregulate extracellular enzyme production when inorganic nutrients are abundant, favouring direct uptake of simpler substrates [55, 56]. Together, our results highlight that microbial metabolism is influenced by both long-term relative nutrient availability constraints and short-term DOM fluctuations [41, 47], though the relative influence of each mechanism likely varies across systems.

### Phosphorus-related pathways and nutrient ratios imbalances

Phosphorus limitation is a key driver of microbial investment into enzymatic hydrolysis and organic P uptake in freshwater systems [5, 57]. Consistent with this, we observed increased representation of P-related predicted pathways under high N:P conditions (Figs 3 and 4), indicating microbial adaptation to P scarcity through phosphatase activity and organic P transport [43, 58]. Similar patterns have been reported in oligotrophic lakes and marine systems [58].

Acid phosphatase, a key enzyme for hydrolyzing organic phosphomonoesters and releasing SRP, showed a positive association with high DIN:SRP ratios, suggesting greater microbial investment in organic P degradation under P-limiting conditions [59, 60]. Its negative correlation with DOC:DIN may reflect shifts in P acquisition strategies under multi-elemental imbalance, or simply interdependence among nutrient ratios [16, 17], i.e. lakes with high DOC:DIN ratios may be unlikely to be P-limited. Interestingly, phosphonate utilization increased under high DOC:DIN conditions, suggesting that these compounds may serve as alternative P sources when N is scarce [61, 62], which is an expected response among N-fixing diazotrophs that rely on phosphonates as alternative to phosphate under N-limited conditions [62].

Despite these relative nutrient availability associations, P-related pathways did not correlate significantly with bioavailable P pools, suggesting that microbial P acquisition is primarily governed by persistent nutrient imbalances rather than short-term fluctuations in labile P availability. This supports previous findings that enzyme production and organic P utilization are typically regulated by long-term P limitation [63, 64].

### Nutrient bioavailability and microbial community composition

Our findings partially supported the hypothesis that bacterial and eukaryotic communities exhibit distinct responses to bioavailable nutrients, reflecting differences in metabolic strategies and nutrient utilization. As expected, eukaryotic communities were more strongly influenced by BDOC than by BTDN or BTDP (Fig. 5D and F), suggesting that labile C plays a central role in structuring their composition, likely through both direct uptake by osmotrophic taxa and indirect effects such as bacterial processing and trophic interactions [65, 66]. In contrast, bacterial communities were more strongly associated with DOC than BDOC (Fig. 5C), which was unexpected given their osmotrophic lifestyle and reliance on dissolved organic compounds for growth [67]. This result suggests that factors beyond immediate C availability influenced the bacterial community structure in our study. For example, the weak influence of BDOC may reflect limitation by other nutrients or by broader nutrient ratios constraints. Previous studies have shown that bacterial composition responds to C:N:P imbalances, with high C:N favouring N limitation and high N:P promoting P limitation [26, 68]. If BDOC is not limiting, bacterial communities may instead be structured by N and P availability, interactions with eukaryotic microbes [21], or access to alternative energy sources such as photoautotrophy [69]. Additionally, total DOC may also influence bacterial communities indirectly by affecting light attenuation, oxygen dynamics, or nutrient availability [34, 70, 71].

For eukaryotic microbes, BDOC responses likely reflect both direct uptake by osmotrophs and indirect trophic interactions. However, the weaker associations with BTDN and BTDP suggests that not all eukaryotes rely directly on dissolved nutrients. Given the dominance of heterotrophs in our samples, prey availability and particulate organic matter may be more important structuring factors, as seen in other systems [72, 73]. Labile C sources such as BDOC can enhance bacterial production, thereby supporting heterotrophic protists and mixotrophs through grazing [66]. Similar patterns have been observed in freshwater systems, where increased DOM availability stimulates bacterial growth, supporting heterotrophic protists and mixotrophs through grazing or nutrient uptake, while also shaping bacterial community composition [34]. Some eukaryotic microbes, including fungi and mixotrophs, can also incorporate dissolved organic compounds directly [65], further explaining the observed relationships with BDOC.

### Trophic strategies and nutrient dynamics in eukaryotic communities

Eukaryotic microbial communities, particularly mixotrophic and heterotrophic, exhibited distinct responses to nutrient gradients. Mixotrophs were more prevalent under N-limited conditions as indicated by their positive correlation with DOC:DIN and negative correlation with DIN:SRP (Fig. 6). Their dual trophic strategy likely confers a competitive advantage by allowing flexible adjustment to resources scarcity [73–76]. Similar trends have been reported in humic lakes, where elevated DOC and declining DIN promote mixotrophic flagellates over heterotrophs, particularly under light limitation caused by browning [77, 78]. In contrast, heterotrophs showed stronger associations with BTDP and high DIN:SRP, suggesting greater dependence on bioavailable fractions of P other than SRP (e.g. bioavailable dissolved organic P) [5, 75, 79]. These patterns underscore the functional diversity within eukaryotic communities, with mixotrophs relying on metabolic flexibility and heterotrophs responding more directly to bioavailable nutrient concentrations.

This divergence in resource use mirrors patterns in bacterial communities, where relative nutrient availability shifts modulate metabolic pathway investment. However, mixotrophs exhibit an added layer of flexibility by integrating photoautotrophic and heterotrophic strategies, distinguishing them from both heterotrophic eukaryotes and osmotrophic bacteria [75, 79]. These findings highlight the ecological importance of microbial trophic diversity in regulating nutrient remineralization and energy flow, emphasizing the need to account for functional group dynamics when evaluating the limitation and ecosystem processes in lakes [3, 80, 81].

## Conclusion

This study addresses a key gap in understanding how microbial communities respond to nutrient availability by jointly examining bioavailable and total or inorganic fractions of C, N, and P in relation to both community composition and metabolic potential. We show that nutrient ratio imbalances are central in structuring microbial communities and functional pathways, whereby bacterial assemblages responded most strongly to DOC concentrations and nutrient ratios, while eukaryotic communities were more influenced by bioavailable nutrients, particularly BDOC and BTDP. Moreover, predicted functional pathways related to N and P metabolism shifted in response to nutrient ratios, reflecting microbial strategies to compensate for limitation through enhanced acquisition, assimilation, or recycling mechanisms. As climate-driven changes continue to alter nutrient supply and organic matter inputs, incorporating microbial metabolic responses into ecosystem models will be essential for improving predictions of nutrient cycling and ecosystem resilience.

## Supplementary Material

Supplementary_material_ycag150

## Data Availability

The 16S and 18S rRNA amplicon sequencing data generated in this study have been deposited in the NCBI Sequence Read Archive under BioProject accession number PRJNA1261026 (link: https://www.ncbi.nlm.nih.gov/bioproject/PRJNA1261026/). The data will be made publicly available upon final acceptance of the manuscript. All other data supporting the findings of this study, including nutrient concentrations, bioassay results, and stoichiometric ratios, are provided in the Supplementary Materials. Analysis scripts are available from the corresponding author upon reasonable request.
