# Supplementary material for: Coupling of nutrient bioavailability and nutrient ratios to microbial community structure and functional potential in lakes
Source: ISME Commun. 2026 May 29;6(1):ycag150. doi: 10.1093/ismeco/ycag150 (PMC13298643; doi:10.1093/ismeco/ycag150)
Supplement: Supplementary_material_ycag150 [file supplementary_material_ycag150.zip › Supplementary_Discussion_final_Rulli_et_al.docx]

Supplementary discussion for **Coupling of nutrient bioavailability and nutrient ratios to microbial community structure and functional potential in lakes**

Running head: **Microbial communities and nutrient ratios**

Mayra P.D. Rulli^1,2^, Romana K. Salis^3^, Ann-Kristin Bergström^4^, Ryan A. Sponseller^4^, Martin Berggren^1^

^1^Department of Physical Geography and Ecosystem Science, Lund University, Sweden

^2^Department of Ecology and Genetics, Uppsala University, Sweden

^3^Department of Biology and Environmental Science, Linnaeus University, Sweden

^4^Department of Ecology, Environment and Geoscience, Umeå University, Sweden

Corresponding author: Mayra Rulli

Mailing address: Norbyvägen 18D, 752 36 Uppsala, Sweden

Email: [rulli.mayra@gmail.com](mailto:rulli.mayra@gmail.com)

**Supplementary discussion**

## Influence of lake characteristics on microbial community composition

Environmental gradients such as lake size and organic matter quality are known to shape microbial communities by influencing nutrient availability and substrate complexity [1, 2]. In our study, bacterial communities were primarily structured by lake size (Fig. S7), consistent with prior research linking physical lake characteristics to microbial diversity and function [1, 3]. Large lakes, with stable nutrient regimes and long residence times, may promote microbial specialization and niche differentiation [1, 4], while smaller lakes experience greater variability and episodic nutrient pulses, favouring flexible taxa capable of coping with fluctuating conditions [4-6].

In contrast, eukaryotic communities were primarily influenced by DOM aromaticity (Fig. S7), with higher SUVA values associated with distinct community structures, potentially signalling changes in both substrate composition and light availability. SUVA reflects the proportion of complex, recalcitrant organic compounds requiring specialized degradation pathways [7, 8]. These compounds may indirectly shape eukaryotic composition by affecting bacterial processing, nutrient regeneration, and microbial food web structure [9, 10]. The strength of this relationship likely varies with functional traits as heterotrophic, mixotrophic, and photoautotrophic eukaryotes differ in their reliance on light, organic matter sources, and microbial prey [10].

# References

1. Lindström, ES, Bergström AK, Influence of inlet bacteria on bacterioplankton assemblage composition in lakes of different hydraulic retention time*.* *Limnology and Oceanography* 2004;**49**:125–36. <https://doi.org/10.4319/lo.2004.49.1.0125>

2. Wang, Y, et al., Spatiotemporal dynamics and determinants of planktonic bacterial and microeukaryotic communities in a Chinese subtropical river*.* *Applied Microbiology and Biotechnology* 2015;**99**:9255–66. <https://doi.org/10.1007/s00253-015-6773-0>

3. Jansson, M, et al., Allochthonous organic carbon and phytoplankton/bacterioplankton production relationships in lakes*.* *Ecology* 2000;**81**:3250–5. <https://doi.org/10.2307/177416>

4. Ruiz-González, C, Niño-García JP, del Giorgio PA, Terrestrial origin of bacterial communities in complex boreal freshwater networks*.* *Ecology Letters* 2015;**18**:1198–206. <https://doi.org/10.1111/ele.12499>

5. Logue, JB, et al., Experimental insights into the importance of aquatic bacterial community composition to the degradation of dissolved organic matter*.* *The ISME Journal* 2016;**10**:533–45. <https://doi.org/10.1038/ismej.2015.131>

6. Jones, RI, Salonen K, Dehaan H, Phosphorus transformations in the epilimnion of humic lakes - abiotic interactions between dissolved humic materials and phosphate*.* *Freshwater Biol.* 1988;**19**:357–69. <https://doi.org/10.1111/j.1365-2427.1988.tb00357.x>

7. Kellerman, AM, et al., Persistence of dissolved organic matter in lakes related to its molecular characteristics*.* *Nature Geoscience* 2015;**8**:454–7. <https://doi.org/10.1038/Ngeo2440>

8. Mostovaya, A, et al., Molecular determinants of dissolved organic matter reactivity in lake water*.* *Frontiers in Earth Science* 2017;**5**:106. <https://doi.org/10.3389/feart.2017.00106>

9. Kellerman, AM, et al., Chemodiversity of dissolved organic matter in lakes driven by climate and hydrology*.* *Nature Communications* 2014;**5**. <https://doi.org/10.1038/Ncomms4804>

10. Mitra, A, et al., Defining planktonic protist functional groups on mechanisms for energy and nutrient acquisition: incorporation of diverse mixotrophic strategies*.* *Protist* 2016;**167**:106–20. <https://doi.org/10.1016/j.protis.2016.01.003>
