# Supplementary material for: Coupling of nutrient bioavailability and nutrient ratios to microbial community structure and functional potential in lakes
Source: ISME Commun. 2026 May 29;6(1):ycag150. doi: 10.1093/ismeco/ycag150 (PMC13298643; doi:10.1093/ismeco/ycag150)
Supplement: Supplementary_material_ycag150 [file supplementary_material_ycag150.zip › Supplementary_Methods_final_Rulli_et_al.docx]

Supplementary methods for **Coupling of nutrient bioavailability and nutrient ratios to microbial community structure and functional potential in lakes**

Running head: **Microbial communities and nutrient ratios**

Mayra P.D. Rulli^1,2^, Romana K. Salis^3^, Ann-Kristin Bergström^4^, Ryan A. Sponseller^4^, Martin Berggren^1^

^1^Department of Physical Geography and Ecosystem Science, Lund University, Sweden

^2^Department of Ecology and Genetics, Uppsala University, Sweden

^3^Department of Biology and Environmental Science, Linnaeus University, Sweden

^4^Department of Ecology, Environment and Geoscience, Umeå University, Sweden

Corresponding author: Mayra Rulli

Mailing address: Norbyvägen 18D, 752 36 Uppsala, Sweden

Email: [rulli.mayra@gmail.com](mailto:rulli.mayra@gmail.com)

# Supplementary Methods

## Bioavailability bioassays and calculation of BDOC, BTDN, and BTDP

Bioavailability assays were conducted using a standardized inoculum created by pooling equal volumes of lake water (n = 34), filtered through a sterile 1.2 µm syringe filter. This approach minimized microbial variability between assays and enabled comparison of nutrient bioavailability based on chemical composition alone.

We followed a bacterial regrowth bioassay modified from [1], adapted from “spike-response” methods [2, 3]. Filtered lake water, growth medium (5% v/v) and inoculum (2% v/v) were combined in 96 deep-well plates and supplemented with C (20 mg C L⁻¹ of glucose, Milipore®), inorganic N (2000 μg N L⁻¹ of NH₄NO₃, Supelco®), and inorganic P (200 µg P L⁻¹ of Na₂HPO₄•2H_2_O, AnalaR NORMAPUR®) solutions to induce nutrient limitation of the targeted nutrient: for C-limited bioassays, N and P solutions were added; for N-limited bioassays, C and P solution were added; and for P-limited bioassays, C and N solutions were added (2% v/v for each solution). For each limitation type, paired spiked wells were amended with an additional spike of the limiting nutrient (C: 1000 µg C L⁻¹; N: 100 µg N L⁻¹; P: 10 µg P L⁻¹). Plates were incubated at 20°C in the dark for 3 days.

One assumption that must be met for successful bioavailability assessment is that strong limitation is induced for the nutrient being essayed. This limitation is required to calculate nutrient assimilation per cell based on the response to nutrient spiking. Moreover, bioassay results must represent the same limiting conditions to be fully comparable. In nearly all incubations, we succeeded to induce the desired limiting conditions for N (except in 2 BTDN essays) and P (except in 1 BTDP essay). However, in as many as 12 BDOC essays, C spikes resulted in absent or slightly negative responses in cell numbers, implying that an unknown factor (not C or other macro- or micronutrients, which we added) was limiting the bacterial growth. Thus, 12, 2, and 1 site for the C, N, and P incubations, respectively, were considered as having missing data when the bioavailability estimation failed (Table S2).

Bacterial abundance was measured at 0, 24, 48, and 72 h using a BD Accuri C6® flow cytometer, after fixation with 3% glutaraldehyde and staining with SYTO 13 [1]. Bacterial growth at 48 hours—corresponding to the primary peak in cell abundance—was used to estimate the concentrations of BDOC, BTDN, and BTDP. These estimates were based on the net cell yield in nutrient-limited incubations, assuming complete assimilation of the added nutrient into biomass, following methods adapted from [2, 3].

First, we calculated the amount of nutrient assimilated per bacterial cell under nutrient-limited conditions (hereafter referred to as nutrient per cell, or Nutrient_cell_) using the following formula: Nutrient_cell_ = s / (A_spike_ −A_non-spike_), where ‘s’ is the concentration of the nutrient added as a spike (μg L^−1^), A_spike_ is the average bacterial abundance in wells with the nutrient spike (cells mL⁻¹), and A_non-spike_ is the average bacterial abundance in wells without the nutrient spike (cells mL⁻¹).

Next, bioavailable nutrient concentrations (B) were calculated by multiplying Nutrient_cell_ estimate by the bacterial abundance in A_non-spike_ wells: B = Nutrient_cell_ * A_non-spike_. This yields the concentration of the bioavailable resource (BDOC, BTDN, and BTDP; in μg L⁻¹) that supported bacterial growth in each lake sample under C-, N-, or P-limited conditions.

## Microbial community analysis

Total DNA was extracted from Sterivex™ filters using the MO BIO PowerSoil DNA isolation kit (MO BIO Laboratories, Carlsbad, CA, USA), following the manufacturer's protocol with modifications for filter-based extractions, and quantified using a Qubit fluorometer (Thermo Fisher Scientific). DNA extraction included six negative controls (two with Milli-Q water, four with no input) to monitor contamination.

Amplicon sequencing of the 16S (V6-V8) and 18S (V4) rRNA gene regions was conducted using an Illumina MiSeq platform with paired-end 2 × 300 bp reads (v3 chemistry, 600 cycles). Primers used were B969F (5'-ACGCGHNRAACCTTACC-3') and BA1406R (5'-ACGGGCRGTGWGTRCAA-3') for 16S, and E572F (5'-CYGCGGTAATTCCAGCTC-3') and E1009R (5'-AYGGTATCTRATCRTCTTYG-3') for 18S [4]. Library preparation followed a single-round PCR approach using fusion primers containing Illumina adaptors, dual indices, and target-specific sequences. PCR amplification was performed in duplicate using separate template dilutions (1:1 and 1:10) with Phusion Plus high-fidelity polymerase (Thermo Fisher Scientific). Each 20 µL PCR reaction contained: 4 µL 5× Phusion Plus Buffer, 0.4 µL dNTPs (40 mM), 4 µL forward primer (1 µM), 4 µL reverse primer (1 µM), 0.2 µL Phusion Plus polymerase (2 U/µL), 5.4 µL PCR-grade water, and 2 µL template DNA. PCR cycling conditions were: initial denaturation at 98°C for 30 s; 30 cycles of 98°C for 10 s, 55°C for 30 s, 72°C for 30 s; final extension at 72°C for 10 min. PCR products were verified on 1% agarose gels, normalized using SequalPrep^™^ Normalization Plate Kit (Thermo Fisher Scientific), and pooled in equimolar concentrations. The final library pool was sequenced on one MiSeq run.

Raw demultiplexed 16S and 18S rRNA sequences were processed with the DADA2 pipeline (v1.30). Primers were removed using ‘cutadapt’ (v4.7), and sequences were filtered and trimmed (parameters: maxN = 0, maxEE = (2,2), truncQ = 2, truncLen = (275,200), rm.phix = TRUE). Sequences were dereplicated, error-corrected, merged, and chimeras were removed. Contaminant ASVs were identified using the decontam package (v1.22), using a prevalence-based approach (threshold = 0.1). ASVs were retained only if classified as bacteria (16S) or eukaryotes (18S). Sequencing yielded an average of 63,994 ± 30,442 raw reads per sample for 16S (range: 15,968–162,524) and 69,550 ± 33,689 raw reads per sample for 18S (range: 24,297–152,608). Following quality filtering, read merging, chimera removal, and contaminant filtering, final datasets contained 40,435 ± 27,546 reads per sample for 16S (range: 5,689–95,996; 63.2% of raw reads retained) and 54,048 ± 28,080 reads per sample for 18S (range: 16,715–116,821; 77.7% of raw reads retained). Complete per-sample sequencing statistics are provided in Table S2. Final ASV tables contained 1,286,321 16S sequences (13,102 ASVs) and 1,779,140 18S sequences (11,585 ASVs).

Eukaryotic ASVs were grouped into trophic categories (phototrophs, heterotrophs, mixotrophs, or unknown) based on taxonomic assignment. First, the Mixoplankton Database (MDB, DOI: 10.5281/zenodo.7560582) was used to identify potential mixotrophic species at the genus or species level. For taxa not found in MDB or identified only to higher taxonomic levels, trophic groups were assigned according to the higher-level taxonomic classification (see complete classification scheme in Table S2), based on the established general understanding of feeding modes in these lineages. For example, Chlorophyta and Bacillariophyceae were classified as phototrophs; Ciliophora and Rhizaria as heterotrophs; and Cryptophyta and Dinoflagellata as mixotrophs. ASVs that could not be confidently assigned to a trophic group were classified as "unknown." ASV tables were CLR-transformed prior to statistical analyses to account for compositionality.

To infer functional potential, we applied PICRUSt2 [5] to 16S rRNA ASVs (filtered, non-normalized counts), predicting gene and pathway abundance based on phylogenetic placement. KEGG Orthology (KO) identifiers were used to annotate metabolic functions. Pathways were grouped into nutrient categories (C, N, P, Fe/metals). Predicted metabolic pathways were analyzed at two levels: individual KEGG-annotated metabolic pathways representing distinct biochemical processes related to four nutrient categories, referred to as specific pathways (shown in Fig. 4); and general pathways, which represent the sum of specific KEGG pathways associated with each nutrient category (shown in Fig. 3).

## Statistical analyses

All analyses were conducted in R (version 2024.12.1). We used the following packages: ‘rstatix’ (v0.7.2) for pairwise comparisons, ‘vegan’ (v2.6-8) for ordination and PERMANOVA, ‘compositions’ (v2.0-8) for CLR transformation, ‘microViz’ for correlation analysis, and ‘ggplot2’ (v3.5.1) for visualisation.

Linear regressions assessed relationships between bioavailable and total or inorganic nutrient concentrations. Dunn’s test with Bonferroni correction was used to compare nutrient ratios. NMDS ordinations were based on Aitchison distance, and PERMANOVA was used to test the relationship between environmental drivers (DOC, DIN, SRP, BDOC, BTDN, BTDP, SUVA, lake size) and bacterial and eukaryotic community composition. PERMANOVAs were performed using the adonis2 function, with Aitchison distance as the dissimilarity metric and 9999 permutations. The full model included all measured nutrient fractions (total DOC, inorganic DIN, SRP, BDOC, BTDN, BTDP), SUVA, and lake size, while additional models were run to evaluate the effects of total or inorganic nutrients (total DOC, inorganic DIN, SRP, SUVA, and lake size), bioavailable nutrients (BDOC, BTDN, BTDP, SUVA, and lake size), and specific C, N, and P fractions. See Table S7 for complete model details and results. Lake size and SUVA were selected as representative modifiers of DOM and physical lake characteristics. A full analysis of lake morphometry and chemistry is beyond the scope of this study.

Distance-based redundancy analysis (db-RDA) with Aitchison distance was applied as a complementary constrained ordination to quantify the influence of environmental variables, db-RDA was applied separately for 16S and 18S data, and each nutrient type was analysed using two model variations: model 1 included lake size and SUVA as covariates, in addition to the specific nutrient pair; model 2 included only the nutrient pair to assess direct nutrient effects. The significance of explanatory variables was assessed using permutation tests with 9999 permutations. See Table S6 for the complete model details and results.

Heatmaps were generated from Spearman correlations between nutrient variables (including log-transformed stoichiometric ratios) and the relative abundances of microbial metabolic pathways or trophic groups. False discovery rate (FDR) correction was applied (Benjamini-Hochberg method), except for the bacterial general pathways analysis.

# References

1. Rulli, MPD, et al., Seasonal patterns in nutrient bioavailability in boreal headwater streams*.* *Limnology and Oceanography* 2022;**67**:1169–83. <https://doi.org/10.1002/lno.12064>

2. Soares, ARA, et al., New insights on resource stoichiometry: assessing availability of carbon, nitrogen, and phosphorus to bacterioplankton*.* *Biogeosciences* 2017;**14**:1527–39. <https://doi.org/10.5194/bg-14-1527-2017>

3. Stepanauskas, R, et al., Summer inputs of riverine nutrients to the Baltic Sea: Bioavailability and eutrophication relevance*.* *Ecological Monographs* 2002;**72**:579–97. <https://doi.org/10.2307/3100058>

4. Comeau, AM, et al., Arctic Ocean microbial community structure before and after the 2007 record sea ice minimum*.* *PloS one* 2011;**6**:e27492.

5. Douglas, GM, et al., PICRUSt2 for prediction of metagenome functions*.* *Nature Biotechnology* 2020;**38**:685–8. <https://doi.org/10.1038/s41587-020-0548-6>
