# Supplementary material for: Coupling of nutrient bioavailability and nutrient ratios to microbial community structure and functional potential in lakes
Source: ISME Commun. 2026 May 29;6(1):ycag150. doi: 10.1093/ismeco/ycag150 (PMC13298643; doi:10.1093/ismeco/ycag150)
Supplement: Supplementary_material_ycag150 [file supplementary_material_ycag150.zip › Supplementary_Results_final_Rulli_et_al.docx]

Supplementary results for **Coupling of nutrient bioavailability and nutrient ratios to microbial community structure and functional potential in lakes**

Running head: **Microbial communities and nutrient ratios**

Mayra P.D. Rulli^1,2^, Romana K. Salis^3^, Ann-Kristin Bergström^4^, Ryan A. Sponseller^4^, Martin Berggren^1^

^1^Department of Physical Geography and Ecosystem Science, Lund University, Sweden

^2^Department of Ecology and Genetics, Uppsala University, Sweden

^3^Department of Biology and Environmental Science, Linnaeus University, Sweden

^4^Department of Ecology, Environment and Geoscience, Umeå University, Sweden

Corresponding author: Mayra Rulli

Mailing address: Norbyvägen 18D, 752 36 Uppsala, Sweden

Email: [rulli.mayra@gmail.com](mailto:rulli.mayra@gmail.com)

# Supplementary Results

**Table S1.** Characteristics of the 34 study lakes, including their location (latitude and longitude), water colour, SUVA (specific ultraviolet absorbance), and chlorophyll-*a* (chl-*a*) concentrations.

| Lakes (sites) | ID | Latitude | Longitude | Water colour  (mg Pt L^−1^) | SUVA  ( L mg^−1^ m^−1^) | Chl-*a*  (µg L^−1^) |
| --- | --- | --- | --- | --- | --- | --- |
| Kallshultasjön | 1 | 56°42'7.13"N | 13°38'52.58"E | 55 | 2.22 | 1.58 |
| Bolmen | 2 | 56°45'42.95"N | 13°39'31.46"E | 56 | 2.24 | 3.09 |
| Vidöstern | 3 | 56°56'59.35"N | 13°58'49.66"E | 60 | 2.28 | 2.91 |
| Furen | 4 | 57°4'32.41"N | 14°10'2.28"E | 61 | 2.31 | 5.83 |
| Rymmen | 5 | 57°5'23.21"N | 14°19'38.35"E | 60 | 2.30 | 5.54 |
| Yasjön | 6 | 57°4'22.69"N | 14°26'3.19"E | 61 | 2.35 | 1.08 |
| Åbodasjön | 7 | 57°4'46.67"N | 14°28'33.35"E | 70 | 2.20 | 1.97 |
| Gyslättasjön | 8 | 57°6'29.05"N | 14°29'17.88"E | 107 | 2.35 | 9.55 |
| Förhultasjön | 9 | 57°7'38.17"N | 14°31'12.68"E | 83 | 2.02 | 3.30 |
| Fiolen | 10 | 57°5'34.19"N | 14°31'47.39"E | 29 | 1.58 | 3.02 |
| Stråken | 11 | 57°3'10.98"N | 14°34'14.77"E | 60 | 2.15 | 1.74 |
| Svanåsasjön | 12 | 57°1'32.77"N | 14°40'39.58"E | 81 | 2.21 | 1.55 |
| Hagesjön | 13 | 56°56'3.59"N | 14°41'17.2"E | 113 | 2.31 | 0.79 |
| Helgasjön | 14 | 56°52'48.94"N | 14°44'13.13"E | 32 | 1.82 | 7.48 |
| Hemmesjösjön | 15 | 56°50'53.2"N | 14°57'9.4"E | 126 | 2.44 | 0.88 |
| Åredasjön | 16 | 56°53'40.09"N | 14°59'3.59"E | 42 | 1.95 | 1.90 |
| Skirsjön | 17 | 56°33'45.65"N | 14°30'43.56"E | 29 | 1.50 | 1.78 |
| Holmasjön | 18 | 57°7'19.49"N | 14°54'56.99"E | 41 | 2.05 | 0.83 |
| Skavenäsasjön | 19 | 57°5'36.17"N | 14°48'13.03"E | 56 | 2.36 | 6.36 |
| Älgarydsjön | 20 | 57°7'1.67"N | 14°45'29.7"E | 81 | 2.11 | 1.72 |
| Hultasjön | 21 | 57°6'32.87"N | 14°42'30.67"E | 49 | 1.99 | 0.61 |
| Klintsjön | 22 | 57°7'23.2"N | 14°41'44.88"E | 103 | 2.08 | 16.21 |
| Värmen | 23 | 57°11'10.68"N | 14°34'53.69"E | 93 | 3.12 | 2.35 |
| Allgunnen | 24 | 57°14'31.6"N | 0°35'5.57"E | 55 | 2.76 | 4.57 |
| Lammen | 25 | 57°10'20.1"N | 14°35'46.28"E | 179 | 3.66 | 4.22 |
| Lången | 26 | 57°10'48.47"N | 14°38'4.27"E | 77 | 2.93 | 3.94 |
| Fersjön | 27 | 57°10'19.49"N | 14°41'44.88"E | 130 | 2.98 | 0.81 |
| Hacksjön | 28 | 57°11'50.17"N | 14°44'59.68"E | 112 | 3.23 | 0.15 |
| Holmeshultasjön | 29 | 57°13'21.47"N | 14°48'50.0"E | 49 | 2.64 | 2.55 |
| Feresjön | 30 | 57°10'10.88"N | 14°48'20.7"E | 50 | 2.78 | 1.32 |
| Skärlen | 31 | 57°9'34.38"N | 14°55'23.66"E | 223 | 3.44 | 1.44 |
| Läen | 32 | 56°43'57.4"N | 15°15'41.87"E | 112 | 2.97 | 0.60 |
| Rolsmosjön | 33 | 56°39'16.45"N | 15°9'25.67"E | 77 | 2.63 | 1.68 |
| Kinnen | 34 | 56°29'11.08"N | 15°12'59.69"E | 231 | 3.16 | 0.45 |

Coordinates correspond to outlet streams sampled to represent the lakes.

**Table S2**. Concentrations of bioavailable, total, and inorganic nutrient concentrations for the 34 study lakes. Missing values of BDOC, BTDN and BTDP represent cases where the bioassay protocol failed to induce C, N and P limitation, respectively, making it impossible to calculate nutrient assimilation per cell based on cell yield responses to nutrient spikes.

| Lakes (sites) | ID | BDOC (mg L^−1^) | BTDN (µg L^−1^) | BTDP (µg L^−1^) | DOC  (mg L^−1^) | DIN  (µg L^−1^) | SRP  (µg L^−1^) |
| --- | --- | --- | --- | --- | --- | --- | --- |
| Kallshultasjön | 1 |  | 122 | 1.51 | 9.19 | 49.5 | 3.8 |
| Bolmen | 2 | 1.5 | 163 | 1.06 | 10.66 | 106 | 3.1 |
| Vidöstern | 3 | 7.4 | 255 | 5.47 | 11.74 | 154.4 | 21.6 |
| Furen | 4 | 3.7 | 132 | 2.66 | 12.55 | 53.1 | 1.8 |
| Rymmen | 5 | 1.3 | 83 | 0.77 | 12.37 | 31.2 | 2.9 |
| Yasjön | 6 | 2.5 | 165 | 2.13 | 11.53 | 48.6 | 2.6 |
| Åbodasjön | 7 | 22.2 | 119 | 1.82 | 14.04 | 46.1 | 4.4 |
| Gyslättasjön | 8 |  | 97 | 7.28 | 15.94 | 45.3 | 1.9 |
| Förhultasjön | 9 | 5 | 1482 | 11.06 | 14.73 | 248.1 | 6.1 |
| Fiolen | 10 |  | 121 | 8.73 | 7.10 | 74.9 | 3.9 |
| Stråken | 11 | 1.8 | 175 | 4.57 | 12.23 | 61.4 | 2.6 |
| Svanåsasjön | 12 | 9.9 | 1066 | 1.67 | 15.27 | 144.9 | 2.7 |
| Hagesjön | 13 |  | 209 | 2.25 | 19.50 | 118.2 | 3.8 |
| Helgasjön | 14 | 2.3 | 36 | 1.52 | 9.85 | 24.1 | 3.6 |
| Hemmesjösjön | 15 |  | 189 | 4.49 | 19.14 | 55.1 | 4.2 |
| Åredasjön | 16 | 2.2 | 138 | 1.69 | 11.01 | 37.8 | 2.6 |
| Skirsjön | 17 | 2.1 | 57 | 0.55 | 12.87 | 37.8 | 3.5 |
| Holmasjön | 18 | 2.1 | 211 | 3.81 | 7.58 | 62.2 | 4 |
| Skavenäsasjön | 19 | 5 | 105 | 2.75 | 10.38 | 53.9 | 2.7 |
| Älgarydsjön | 20 |  | 255 | 8.39 | 14.53 | 101.6 | 4.4 |
| Hultasjön | 21 | 6.3 | 1347 | 2.06 | 9.22 | 1132.4 | 4.8 |
| Klintsjön | 22 |  | 81 | 41.24 | 19.76 | 2209.2 | 12.8 |
| Värmen | 23 | 2.8 | 328 | 0.8 | 12.99 | 103.8 | 4.8 |
| Allgunnen | 24 | 5.8 | 112 | 0.87 | 10.23 | 46.4 | 5.5 |
| Lammen | 25 | 3.1 | 70 | 0.97 | 17.56 | 33.6 | 5.1 |
| Lången | 26 | 3.8 | 39 | 2.15 | 11.57 | 24 | 4.2 |
| Fersjön | 27 |  | 349 | 2.8 | 16.56 | 151.7 | 4 |
| Hacksjön | 28 | 5.2 | 1302 | 1.95 | 12.39 | 93.8 | 2.6 |
| Holmeshultasjön | 29 | 4.7 | 94 | 0.26 | 9.97 | 37.2 | 4.8 |
| Feresjön | 30 | 4.3 | 172 | 1.18 | 7.55 | 48.2 | 4.1 |
| Skärlen | 31 |  |  |  | 21.33 | 156.4 | 4.8 |
| Läen | 32 |  | 514 | 8.76 | 15.04 | 1204.1 | 41.2 |
| Rolsmosjön | 33 |  | 680 | 6.18 | 10.98 | 265.6 | 3.6 |
| Kinnen | 34 |  |  | 6.45 | 27.43 | 385 | 4.6 |

**Table S3.** Sequencing sample data. Number of raw sequences reads for each sample (lake), number of reads obtained after quality filtering, merging, chimera removal and the removal of contaminants.

|  | 16S rRNA | | | | | 18S rRNA | | | | |
| --- | --- | --- | --- | --- | --- | --- | --- | --- | --- | --- |
| Lake ID | Raw | Filtered | Merged | Chimera | Final | Raw | Filtered | Merged | Chimera | Final |
| 1 | 117136 | 88811 | 80124 | 77131 | 75489 | 56245 | 45896 | 39282 | 39041 | 38910 |
| 2 | 101417 | 76057 | 70177 | 67652 | 64362 | 68059 | 56024 | 52819 | 52546 | 52457 |
| 3 | 111967 | 83864 | 75176 | 70665 | 68168 | 61514 | 49576 | 43679 | 43417 | 42748 |
| 4 | 128958 | 94519 | 84038 | 79552 | 77177 | 79411 | 64916 | 60540 | 58736 | 58564 |
| 5 | 162524 | 118827 | 108445 | 103884 | 95996 | 103183 | 84894 | 78399 | 77070 | 76877 |
| 6 | 108148 | 79905 | 69519 | 68338 | 61680 | 69509 | 55735 | 50743 | 50662 | 50578 |
| 7 | 87888 | 65440 | 59207 | 58648 | 54541 | 73213 | 59564 | 56815 | 56703 | 56452 |
| 8 | 67065 | 50342 | 43660 | 42576 | 41746 | 39060 | 31013 | 29564 | 29156 | 29136 |
| 9 | 89293 | 66301 | 48280 | 46061 | 43012 | 46662 | 37936 | 33299 | 31710 | 31661 |
| 10 | 111125 | 83534 | 67288 | 64696 | 63704 | 24297 | 20034 | 16875 | 16807 | 16715 |
| 11 | 106040 | 79554 | 70509 | 69161 | 63938 | 37547 | 30845 | 26453 | 26435 | 26312 |
| 12 | 103330 | 77115 | 70701 | 69655 | 67060 | 31143 | 25340 | 22946 | 22915 | 22820 |
| 13 | 114913 | 84339 | 71962 | 69407 | 67448 | 39660 | 32529 | 30138 | 30113 | 29775 |
| 14 | 115906 | 87956 | 81752 | 78640 | 70240 | 37715 | 31114 | 28489 | 28472 | 28374 |
| 15 | 61384 | 46469 | 38879 | 38034 | 37151 | 25053 | 21058 | 19591 | 19426 | 19363 |
| 16 | 15968 | 11913 | 9995 | 9815 | 9432 | 55333 | 46168 | 43363 | 43317 | 43178 |
| 17 | 26340 | 19689 | 18584 | 18233 | 17312 | 81363 | 67253 | 64517 | 64323 | 64265 |
| 18 | 31175 | 23497 | 19275 | 18983 | 17835 | 127996 | 105959 | 100495 | 100331 | 99568 |
| 19 | 64271 | 48335 | 41879 | 41253 | 34770 | 90118 | 74150 | 68782 | 68549 | 68326 |
| 20 | 66683 | 50520 | 46366 | 45670 | 43923 | 139265 | 118264 | 115869 | 115539 | 115406 |
| 21 | 38948 | 28794 | 21621 | 20859 | 20544 | 86484 | 70335 | 65291 | 64124 | 63996 |
| 22 | 30350 | 23232 | 19978 | 18930 | 16803 | 49882 | 42281 | 40528 | 40479 | 40090 |
| 23 | 38643 | 29353 | 26398 | 25811 | 24006 | 77330 | 63689 | 59229 | 58226 | 57931 |
| 24 | 27660 | 21111 | 19743 | 19323 | 18623 | 38686 | 32003 | 29093 | 27314 | 27228 |
| 25 | 25666 | 19347 | 16839 | 16652 | 12780 | 58355 | 48150 | 44980 | 44113 | 44097 |
| 26 | 20128 | 15338 | 13694 | 13208 | 10615 | 84822 | 71454 | 67813 | 66698 | 66600 |
| 27 | 32687 | 24011 | 19761 | 19367 | 18708 | 152608 | 122549 | 117644 | 116991 | 116821 |
| 28 | 50988 | 36763 | 26254 | 25507 | 24560 | 67014 | 56381 | 52955 | 52746 | 52615 |
| 29 | 41302 | 30890 | 26064 | 25553 | 22025 | 140983 | 116119 | 110987 | 110351 | 110018 |
| 30 | 23363 | 16608 | 11739 | 11380 | 9801 | 48147 | 39821 | 35778 | 35458 | 35411 |
| 31 | 21210 | 12307 | 6037 | 5761 | 5689 | 55739 | 45409 | 38185 | 37692 | 37632 |
| 32 | 20024 | 14498 | 7858 | 7657 | 7252 | 47576 | 37115 | 30287 | 30148 | 30122 |
| 33 | 20743 | 14928 | 7745 | 7444 | 7329 | 65097 | 52926 | 46697 | 46269 | 42179 |
| 34 | 35790 | 25973 | 13394 | 12816 | 12602 | 117487 | 95914 | 84665 | 84031 | 82915 |

**Table S4.** Taxonomic criteria for eukaryotic trophic group classification. Eukaryotic ASVs identified from 18S rRNA gene amplicon sequencing were assigned to trophic groups based on their taxonomy and primary mode of energy acquisition. First, the Mixoplankton Database (MDB, DOI: 10.5281/zenodo.7560582) was used to identify potential mixotrophic species at the genus or species level. For taxa not found in MDB or identified only to higher taxonomic levels, trophic groups were assigned according to the taxonomic classification scheme shown below, based on the established general understanding of potential feeding modes in these lineages.

| **Trophic Group** | **Taxonomic Level** | **Taxa assigned** |
| --- | --- | --- |
| Phototroph | Division | Chlorophyta, Rhodophyta, Streptophyta |
|  | Subdivision | Chrompodellids |
|  | Class | Bacillariophyceae, Bolidophyceae, Coscinodiscophyceae, Eustigmatophyceae, Mediophyceae, Phaeothamniophyceae, Phaeophyceae, Raphidophyceae, Xanthophyceae |
| Heterotroph | Supergroup | Amoebozoa, CRuMs, Obazoa, Provora |
|  | Division | Kathablepharidacea, Ancyromonadida, Hemimastigophora, Centroplasthelida, Rhizaria, Telonemia |
|  | Subdivision | Discoba_X, Apicomplexa, Ciliophora, Colponemidia, Perkinsea, Bigyra |
|  | Class | Gyrista_X, Hyphochytriomyceta, Peronosporomycetes, Pirsoniales |
| Mixotroph | Division | Haptophyta, Cryptophyta |
|  | Subdivision | Dinoflagellata, Euglenozoa |
|  | Class | Chrysophyceae |

**Table S5**. Bioavailable and total or inorganic resource ratios for the 34 study lakes.

| Lakes (sites) | ID | BDOC:  BTDN | BDOC:  BTDP | BTDN:  BTDP | DOC:  DIN | DOC:  SRP | DIN:  SRP |
| --- | --- | --- | --- | --- | --- | --- | --- |
| Kallshultasjön | 1 |  |  | 179 | 217 | 6236 | 28.80 |
| Bolmen | 2 | 10.48 | 3564 | 340 | 117 | 8867 | 75.59 |
| Vidöstern | 3 | 33.99 | 3503 | 103 | 88.70 | 1402 | 15.80 |
| Furen | 4 | 32.27 | 3540 | 110 | 276 | 17979 | 65.21 |
| Rymmen | 5 | 18.82 | 4484 | 238 | 462 | 10999 | 23.78 |
| Yasjön | 6 | 17.63 | 3019 | 171 | 277 | 11435 | 41.32 |
| Åbodasjön | 7 | 217 | 31436 | 145 | 355 | 8228 | 23.16 |
| Gyslättasjön | 8 |  |  | 29 | 410 | 21634 | 52.70 |
| Förhultasjön | 9 | 3.94 | 1168 | 296 | 69.26 | 6227 | 89.91 |
| Fiolen | 10 |  |  | 30.64 | 111 | 4695 | 42.45 |
| Stråken | 11 | 11.79 | 998 | 84.65 | 232 | 12130 | 52.20 |
| Svanåsasjön | 12 | 10.78 | 15214 | 1411 | 123 | 14584 | 119 |
| Hagesjön | 13 |  |  | 205 | 192 | 13233 | 68.76 |
| Helgasjön | 14 | 75.82 | 3970 | 52.36 | 477 | 7056 | 14.80 |
| Hemmesjösjön | 15 |  |  | 93.05 | 405 | 11751 | 29.00 |
| Åredasjön | 16 | 18.50 | 3339 | 181 | 340 | 10920 | 32.14 |
| Skirsjön | 17 | 43.78 | 10029 | 229 | 397 | 9482 | 23.87 |
| Holmasjön | 18 | 11.85 | 1451 | 122 | 142 | 4887 | 34.37 |
| Skavenäsasjön | 19 | 55.25 | 4663 | 84.40 | 225 | 9914 | 44.13 |
| Älgarydsjön | 20 |  |  | 67.19 | 167 | 8516 | 51.04 |
| Hultasjön | 21 | 5.48 | 7918 | 1445 | 9.50 | 4953 | 522 |
| Klintsjön | 22 |  |  | 4.34 | 10.43 | 3981 | 382 |
| Värmen | 23 | 9.83 | 8906 | 906 | 146 | 6979 | 47.80 |
| Allgunnen | 24 | 60.35 | 17173 | 285 | 257 | 4796 | 18.65 |
| Lammen | 25 | 51.63 | 8236 | 160 | 610 | 8879 | 14.56 |
| Lången | 26 | 113 | 4549 | 40.10 | 562 | 7104 | 12.63 |
| Fersjön | 27 |  |  | 276 | 127 | 10676 | 83.84 |
| Hacksjön | 28 | 4.70 | 6935 | 1476 | 154 | 12288 | 79.75 |
| Holmeshultasjön | 29 | 58.41 | 46684 | 799 | 313 | 5356 | 17.13 |
| Feresjön | 30 | 29.20 | 9408 | 322 | 183 | 4749 | 25.99 |
| Skärlen | 31 |  |  |  | 159 | 11459 | 72.03 |
| Läen | 32 |  |  | 130 | 14.57 | 941 | 64.61 |
| Rolsmosjön | 33 |  |  | 243 | 48.22 | 7865 | 163 |
| Kinnen | 34 |  |  |  | 83.11 | 15377 | 185 |

**Table S6** Results of redundancy analysis (RDA) examining the relationships between microbial community composition (16S rRNA and 18S rRNA) and environmental variables across study lakes. The table presents RDA models for bacterial and eukaryotic communities, with separate models focusing on phosphorus (P), nitrogen (N), and carbon (C), as well as full models incorporating multiple nutrient fractions. Each nutrient type is analysed using two model variations: model 1 includes lake size and SUVA as covariates, in addition to the specific nutrient pair; model 2 includes only the nutrient pair to assess direct nutrient effects. For each model raw variance, % variance explained, F-value, and p-value are reported. Df is equal to 1 for all factors in each model. Significant results (p < 0.05) are bolded.

|  | Bacterial community | | | | Eukaryotic community | | | |
| --- | --- | --- | --- | --- | --- | --- | --- | --- |
|  | Raw variance | % variance explained | F-value | p-value | Raw variance | % variance explained | F-value | p-value |
| *P model 1: community composition ~ SRP + BTDP + SUVA + Lake size* | | | | | | | | |
| SRP | 43.468 | 27.583 | 1.413 | 0.130 | 37.016 | 22.197 | 1.025 | 0.371 |
| BTDP | 28.932 | 18.361 | 0.940 | 0.544 | 63.878 | 38.304 | 1.768 | **0.048** |
| SUVA | 24.949 | 15.833 | 0.811 | 0.854 | 41.298 | 24.761 | 1.143 | 0.169 |
| Lake size | 60.238 | 38.223 | 1.958 | **0.009** | 24.579 | 14.738 | 0.680 | 0.850 |
| *P model 2: community composition ~ SRP + BTDP* | | | | | | | | |
| SRP | 44.874 | 60.938 | 1.42 | 0.135 | 34.921 | 34.255 | 0.972 | 0.408 |
| BTDP | 28.765 | 39.062 | 0.911 | 0.569 | 67.022 | 65.745 | 1.866 | **0.036** |
| *N model 1: community composition ~ DIN + BTDN + SUVA + Lake size* | | | | | | | | |
| DIN | 31.563 | 21.300 | 0.972 | 0.524 | 56.012 | 33.845 | 1.564 | 0.057 |
| BTDN | 30.426 | 20.533 | 0.937 | 0.563 | 45.271 | 27.353 | 1.264 | 0.086 |
| SUVA | 25.177 | 16.990 | 0.775 | 0.901 | 38.285 | 23.133 | 1.069 | 0.282 |
| Lake size | 61.019 | 41.177 | 1.879 | **0.022** | 25.924 | 15.660 | 0.724 | 0.817 |
| *N model 2: community composition ~ DIN + BTDN* | | | | | | | | |
| DIN | 32.054 | 50.063 | 0.965 | 0.527 | 57.337 | 55.709 | 1.612 | 0.053 |
| BTDN | 31.962 | 49.937 | 0.962 | 0.514 | 45.579 | 44.291 | 1.282 | 0.073 |
| *C model 1: community composition ~ DOC + BDOC + SUVA + Lake size* | | | | | | | | |
| DOC | 60.16 | 26.387 | 1.368 | **0.048** | 57.657 | 22.814 | 1.046 | 0.352 |
| BDOC | 42.43 | 18.610 | 0.965 | 0.577 | 102.777 | 40.674 | 1.864 | **0.001** |
| SUVA | 58.98 | 25.870 | 1.341 | 0.061 | 50.492 | 19.982 | 0.916 | 0.709 |
| Lake size | 66.390 | 29.133 | 1.510 | 0.080 | 41.804 | 16.541 | 0.758 | 0.809 |
| *C model 2: community composition ~ DOC + BDOC* | | | | | | | | |
| DOC | 63.999 | 57.245 | 1.396 | **0.049** | 58.243 | 35.330 | 1.075 | 0.296 |
| BDOC | 47.792 | 42.755 | 1.043 | 0.475 | 106.602 | 64.670 | 1.967 | **0.001** |

**Table S7** Results of permutational multivariate analysis of variance (PERMANOVA) testing the influence of environmental variables on microbial community composition. PERMANOVA was conducted separately for bacterial (16S rRNA) and eukaryotic (18S rRNA) communities, using Aitchison distance as the dissimilarity metric and 9999 permutations. All continuous variables were centred and scaled prior to analysis. Each row presents results from models testing different sets of environmental predictors. The full model included all measured nutrient fractions (total DOC, inorganic DIN, SRP, BDOC, BTDN, BTDP), SUVA, and lake size. Additional models were run to evaluate the effects of total or inorganic nutrients, bioavailable nutrients, and specific C, N, and P fractions. The model structure is shown in each section of the table. R² values indicate the proportion of variance explained by each factor, and p-values denote statistical significance. Df is equal 1 for all factors in each model. Significant results (p < 0.05) are bolded.

|  | Bacterial communities | | | Eukaryotic communities | | |
| --- | --- | --- | --- | --- | --- | --- |
| Factor | F-value | r^2^ | p-value | F-value | r^2^ | p-value |
| *Full model: community composition ~ DOC + DIN + SRP + BDOC + BTDN + BTDP + SUVA + Lake size* | | | | | | |
| DOC | 0.738 | 0.031 | 0.822 | 1.174 | 0.052 | 0.217 |
| DIN | 1.739 | 0.073 | **0.050** | 1.836 | 0.082 | **0.040** |
| SRP | 1.536 | 0.064 | 0.122 | 0.838 | 0.037 | 0.579 |
| BDOC | 1.402 | 0.059 | 0.178 | 0.664 | 0.030 | 0.874 |
| BTDN | 1.031 | 0.043 | 0.401 | 1.520 | 0.068 | 0.069 |
| BTDP | 0.763 | 0.032 | 0.796 | 1.061 | 0.047 | 0.321 |
| SUVA | 0.812 | 0.034 | 0.706 | 1.140 | 0.051 | 0.240 |
| Lake size | 2.667 | 0.111 | **0.003** | 0.941 | 0.042 | 0.379 |
| *Total or inorganic model: community composition ~ DOC + DIN + SRP + SUVA + Lake size* | | | | | | |
| DOC | 1.426 | 0.040 | **0.049** | 1.035 | 0.031 | 0.347 |
| DIN | 1.201 | 0.034 | 0.245 | 1.375 | 0.041 | 0.112 |
| SRP | 1.396 | 0.040 | 0.135 | 1.075 | 0.032 | 0.338 |
| SUVA | 0.819 | 0.023 | 0.821 | 1.304 | 0.039 | 0.066 |
| Lake size | 2.491 | 0.070 | **0.001** | 0.802 | 0.024 | 0.598 |
| *Bioavailable model: community composition ~ BDOC + BTDN + BTDP + SUVA + Lake size* | | | | | | |
| BDOC | 1.386 | 0.061 | 0.186 | 0.659 | 0.031 | 0.895 |
| BTDN | 1.315 | 0.058 | 0.149 | 1.575 | 0.073 | **0.044** |
| BTDP | 0.835 | 0.037 | 0.654 | 1.085 | 0.050 | 0.293 |
| SUVA | 0.782 | 0.035 | 0.767 | 1.355 | 0.063 | 0.091 |
| Lake size | 2.428 | 0.107 | **0.007** | 0.886 | 0.041 | 0.458 |
| *C model: community composition ~ DOC + BDOC + SUVA + Lake size* | | | | | | |
| DOC | 0.722 | 0.032 | 0.882 | 1.015 | 0.049 | 0.392 |
| BDOC | 1.286 | 0.058 | 0.221 | 0.682 | 0.033 | 0.893 |
| SUVA | 0.742 | 0.033 | 0.874 | 1.249 | 0.060 | 0.125 |
| Lake size | 2.425 | 0.109 | **0.007** | 0.843 | 0.040 | 0.521 |
| *N model: community composition ~ DIN + BTDN + SUVA + Lake size* | | | | | | |
| DIN | 1.028 | 0.032 | 0.390 | 1.622 | 0.050 | 0.066 |
| BTDN | 0.982 | 0.030 | 0.453 | 1.496 | 0.046 | **0.045** |
| SUVA | 0.842 | 0.026 | 0.728 | 1.544 | 0.048 | **0.023** |
| Lake size | 2.481 | 0.076 | **0.007** | 0.793 | 0.024 | 0.603 |
| *P model: community composition ~ SRP + BTDP + SUVA + Lake size* | | | | | | |
| SRP | 1.318 | 0.039 | 0.195 | 1.059 | 0.032 | 0.318 |
| BTDP | 1.101 | 0.033 | 0.309 | 1.667 | 0.050 | **0.064** |
| SUVA | 1.064 | 0.031 | 0.327 | 1.641 | 0.049 | **0.014** |
| Lake size | 2.438 | 0.073 | **0.003** | 0.787 | 0.024 | 0.621 |

PERMANOVA analyses confirmed that lake size significantly explained bacterial community composition on the full model (r² = 0.11, p < 0.003) as well as in all other tested models (Table S7). However, SUVA was not significant in the full model but was significant in models that included specific nutrient fractions. Specifically, SUVA was a significant predictor of eukaryotic community composition in the P-focused model (r^2^ = 0.05, p = 0.014; Table S7), while BTDN and SUVA were significant in the N-focused model (r^2^ = 0.05, p = 0.045 and 0.023, respectively; Table S7). Moreover, BTDN was significant when only bioavailable fractions were considered (r^2^ = 0.073, p = 0.044; Table S7) and DOC was a significant predictor of bacterial community composition when only total nutrients were considered (r^2^ = 0.04, p = 0.049).


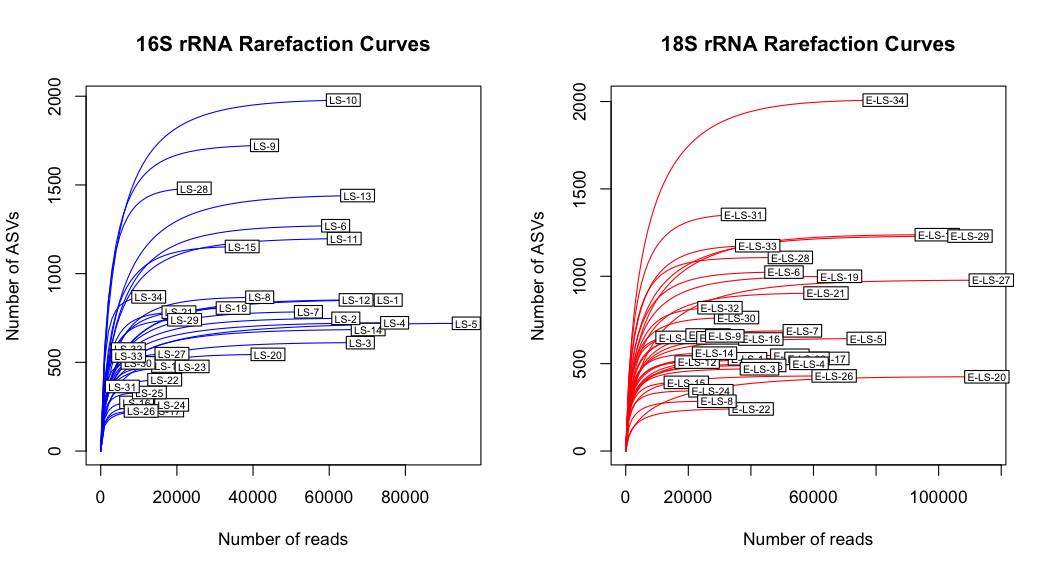


**Figure S1** Rarefaction curves for the 16S rRNA and 18S rRNA gene amplicon sequencing data (post-filtering).

**Figure S2** Functional relative abundance across lakes. Bars represent the relative abundance of predicted functional pathways in each lake, with colours indicating general pathway categories analysed in this study.

**Figure S3** Heatmap of the Spearman’s rank correlations displaying the correlation coefficient values (*rho*) between predicted general functional pathways and environmental variables (BDOC, DOC, BTDN, DIN, BDTP, SRP, Chl-*a*, and Lake size). Colour intensity reflects the strength and direction of the correlation (red = positive, blue = negative), with asterisks indicating statistical significance (*p < 0.05, not FDR-adjusted). The boxplot shows the log10-transformed abundance for each functional pathway.

**Figure S4** Heatmap of the Spearman’s rank correlations displaying the correlation coefficient values (*rho*) between the selected predicted specific functional pathways and environmental variables (BDOC, DOC, BTDN, DIN, BDTP, SRP, Chl-*a*, and Lake size). Colour intensity reflects the strength and direction of the correlation (red = positive, blue = negative), with asterisks indicating statistical significance (*p < 0.05, **p < 0.01, not FDR-adjusted). The boxplot shows the log10-transformed abundance for each functional pathway.

SUVA and lake size showed functional associations, with SUVA positively correlated with fermentation pathways (p = 0.03, *rho* = 0.47; Fig. S4), while lake size was negatively correlated with iron-chelating compounds and nitrite reductases (p = 0.02 and 0.008, respectively; Fig. S4).

**Figure S5** Trophic relative abundance across lakes. Bars represent the relative abundance of trophic groups in each lake, with colours indicating different trophic classifications.

# Figure S6 Heatmap of the Spearman’s rank correlations displaying the correlation coefficient values (*rho*) between trophic group (phototroph, mixotroph, heterotroph, unknown) and environmental variables (BDOC, DOC, BTDN, DIN, BDTP, SRP, Chl-*a*, and Lake size). Colour intensity reflects the strength and direction of the correlation (red = positive, blue = negative), with asterisks indicating statistical significance (*p < 0.05, FDR-adjusted). The boxplot shows the log10-transformed abundance for each trophic group.

**Figure S7** A non-metric multidimensional scaling (NMDS) ordination of A) bacterial and B) eukaryotic microbial communities based on 16S and 18S rRNA amplicon sequencing, respectively. The ordination is based on Aitchison distance. Lake size and SUVA (variables with p < 0.05) are represented as vectors. The length and direction of the vectors indicate the strength and orientation of their correlation with microbial community composition. Stress values for NMDS1 = 0.19 and 0.15 for panels A and B, respectively.

Non-metric multidimensional scaling (NMDS) ordination revealed distinct structuring of bacterial and eukaryotic microbial communities in relation to lake characteristics and nutrient availability (Fig. S7). Bacterial communities were moderately structured by lake size (r^2^ = 0.35, p = 0.047; Fig. S7-A). In contrast, eukaryotic communities were more influenced by SUVA (r^2^ = 0.33, p = 0.027; Fig. S7-B), showing gradual compositional shifts along the gradient of DOM aromaticity. Lake size and SUVA were the only significant variables for bacterial and eukaryotic communities, respectively.
